# 598 Structural Characterization and Safety Validation of Cultured Skin Cells in Pediatric Incubators: A Preliminary Report

**DOI:** 10.1093/jbcr/irae036.232

**Published:** 2024-04-17

**Authors:** Wayne G Kleintjes, Tarryn K Prinsloo

**Affiliations:** Stellenbosch University, Cape Town, Western Cape; Cape Peninsula University of Technology/Biosigns Therapeutics, Cape Town, Western Cape; Stellenbosch University, Cape Town, Western Cape; Cape Peninsula University of Technology/Biosigns Therapeutics, Cape Town, Western Cape

## Abstract

**Introduction:**

Skin substitutes, such as cultured epithelial autografts (CEA), have been utilized for wound treatment with improved outcomes. CEA can be used as a ‘stand-alone’ treatment, but combining it with temporary coverage has shown to increase graft take. The aim of this preliminary observational and prospective controlled study was to report on the histological findings of the cultured CEA and to demonstrate its safety for routine use.

**Methods:**

Skin cells were retrieved from biopsies and placed in autologous platelet-rich plasma. The cells were then seeded onto bacteria- and fungi binding dressing pads that is routinely used and incubated in pediatric incubators at 37 °C. Fresh plasma was applied daily and amorphous hydrogel administered every third or fourth day. After 2 weeks, confluence was reached, the culture sample was fixed with to the slide. Hematoxylin and eosin, and Masson’s Trichrome stains were used for histological evaluation. Light microscopy at 4X and 10X magnification was used to observe the cultured epithelial layers and cells, respectively. Bacteriological safety was externally assessed by analyzing wound swabs of the confluent monolayers grown in both pediatric and standard culture incubators (n=10 each) before and after transplant for microscopy and culturing. The primary outcome of was skin histology and the secondary outcome was the presence of pathogens.

**Results:**

Typical epidermal and dermal (papillary and reticular) layers were clearly observed, along with the classical differentiation patterns of the epidermal sublayers. Keratinocytes were observed as densely packed colonies along with the mediated spread of collagen. The potential presence of adipose droplets could not be excluded. No pathogenic organisms were observed in any of the cell culture specimens tested.

**Results:**

Typical epidermal and dermal (papillary and reticular) layers were clearly observed, along with the classical differentiation patterns of the epidermal sublayers. Keratinocytes were observed as densely packed colonies along with the mediated spread of collagen. The potential presence of adipose droplets could not be excluded. No pathogenic organisms were observed in any of the cell culture specimens tested.

**Conclusions:**

The differentiated nature of this dressing-grown CEA emulated the epidermis to a large degree. Cell differentiation was not only indicative of functional integrity, but mechanical integrity outcomes of the technique as well. No difference could be demonstrated between the different incubators in terms of the pathogenic bacteria grown or cell culture growth.

**Applicability of Research to Practice:**

These preliminary findings suggest that the cultivated CEA using the modified technique may possess ideal skin-related characteristics that is safe for use. This is essential for favorable grafting outcomes that is not limited as treatment for burn wounds only.

